# Association between the naturally nutrient rich score and type 2 diabetes mellitus risk: a case–control study

**DOI:** 10.3389/fnut.2026.1889494

**Published:** 2026-08-24

**Authors:** Saad Mohammed Almohya, Nasser Alqahtani

**Affiliations:** 1 Riyadh Health First Cluster, Riyadh, Saudi Arabia; 2 Northern Border University, Arar, Saudi Arabia

**Keywords:** case–control study, dietary quality, naturally nutrient rich score, nutrient density, type 2 diabetes mellitus

## Abstract

**Background:**

The Naturally Nutrient Rich (NNR) score is a validated nutrient-density metric that quantifies micronutrient adequacy relative to energy intake, yet its association with type 2 diabetes mellitus (T2DM) risk remains uninvestigated. This study aimed to examine the association between the NNR score and T2DM risk among Saudi adults.

**Methods:**

A hospital-based case–control study was conducted at Prince Abdulaziz bin Musaed Hospital, Saudi Arabia between November 2024 and August 2025, enrolling 140 confirmed T2DM cases and 280 non-diabetic controls (same healthcare facility) matched 1:2 by age and sex. Dietary intake was assessed using a validated 152-item semi-quantitative food frequency questionnaire. The NNR score was calculated as the mean percentage daily value (%DV) of 14 key nutrients per 2,000 kcal (protein, vitamins A, C, D, E, B1, B2, and B12, calcium, zinc, iron, folate, potassium, and unsaturated fatty acids). Logistic regression models were applied to evaluate the association between the NNR score and T2DM risk, with progressive adjustment for age, sex, smoking, physical activity, BMI, and total caloric intake.

**Results:**

Cases had significantly higher BMI (28.32 ± 2.64 vs. 26.85 ± 2.15 kg/m^2^; *p* = 0.039), caloric intake, carbohydrate, and cholesterol consumption, alongside significantly lower intakes of dietary fibre, PUFA, magnesium, selenium, folate, vitamins A, E, and B₁, beta-carotene, and biotin compared to controls. Participants with higher NNR scores demonstrated significantly greater intakes of fibre, zinc, potassium, magnesium, and multiple B vitamins, without significant differences in total energy or macronutrient intake. Across all logistic regression models, higher NNR scores were independently and inversely associated with T2DM risk, with the fully adjusted model yielding an odds ratio of 0.90 (95% CI: 0.82–0.93; *p* < 0.001).

**Conclusion:**

Higher dietary nutrient density, as measured by the NNR score, is significantly and independently associated with reduced odds of T2DM in Saudi adults. These findings underscore the relevance of micronutrient adequacy, beyond total energy intake, in relation to lower odds of T2DM and support the integration of nutrient density metrics into dietary guidance and public health strategies.

## Introduction

Type 2 diabetes mellitus (T2DM) is a chronic metabolic disorder characterised by progressive insulin resistance and impaired pancreatic beta-cell function, and it has reached epidemic proportions globally. According to the 11th edition of the International Diabetes Federation (IDF) Diabetes Atlas, approximately 589 million adults aged 20–79 years were living with diabetes in 2024 with projections estimating a further rise to 853 million by 2050 (1). The burden is particularly pronounced in the Middle East and North Africa (MENA) region, where the Kingdom of Saudi Arabia (KSA) records one of the highest national prevalence rates in the world. A systematic review and meta-analysis of studies conducted between 2016 and 2022 estimated a pooled T2DM prevalence of 28% among Saudi adults (2), with epidemiological modelling suggesting a trajectory from 8.5% in 1992 to nearly 39.5% by 2022 (3). This alarming rise is closely tied to rapid urbanisation, physical inactivity, rising obesity rates, and profound shifts in dietary behaviour all of which are well-established modifiable risk factors in the pathogenesis of T2DM (4, 5). Among these modifiable determinants, diet has attracted particular attention given its central and reversible role in metabolic risk.

Diet is widely recognised as a central modifiable determinant of T2DM risk, and considerable research has sought to capture the overall quality of habitual dietary intake through composite dietary quality indices (DQIs) such as the Healthy Eating Index (HEI), the Mediterranean Diet Score (MDS), and the Dietary Inflammatory Index (DII), which, respectively, assess adherence to national dietary guidelines, adherence to a specific regional dietary pattern, and the pro−/anti-inflammatory potential of habitual intake. These indices were largely developed and validated in Western populations and depend on adherence to predefined food patterns rather than on nutrient adequacy per se, which may limit their direct applicability to the Saudi dietary context—characterised by a hybrid pattern combining traditional plant-based staples with increasing refined-carbohydrate and processed-food intake. Among these, the Naturally Nutrient Rich (NNR) score, originally proposed by Drewnowski (6), is a validated nutrient-density metric that calculates the mean percentage of daily values (%DV) for 14 key beneficial nutrients including protein, vitamins A, C, D, E, B1, B2, B12, calcium, zinc, iron, folate, potassium, and unsaturated fatty acids per 2,000 kcal of dietary intake. Unlike indices that focus primarily on restricting detrimental nutrients, or on scoring adherence to a specific dietary pattern, such as the HEI or MDS, the NNR score adopts a positive approach by quantifying the adequacy of micronutrient supply relative to energy intake, independent of any single culturally specific dietary pattern, an attribute that may make it more readily generalisable to non-Western populations such as Saudi Arabia, thereby providing a standardised and population-applicable measure of overall dietary quality (6, 7). The NNR score has been validated across diverse populations as a reliable tool for distinguishing nutrient-dense from energy-dense but nutrient-poor diets, and it has demonstrated utility in evaluating diet quality in relation to various health outcomes (8, 9).

The biological rationale linking nutrient-dense diets to reduced T2DM risk is well-supported. Micronutrients central to the NNR score including magnesium, zinc, and vitamins D and B12 play critical roles in insulin signalling, glucose transporter regulation, and pancreatic beta-cell integrity Evidence from predominantly observational studies indicates that diets characterised by high nutrient density are associated with improved insulin sensitivity, reduced systemic inflammation, and more favourable glycaemic profiles (10), although such designs establish association rather than causation. Empirical evidence corroborates these mechanisms: a prospective analysis within the CORDIOPREV cohort found that greater improvements in nutrient-rich food scores were associated with over a 50% reduction in incident T2DM risk (11). A further case–control study employing the Diet Quality Index–International confirmed a protective effect of overall dietary quality on T2DM risk, supporting the relevance of composite nutrition indices in this context (12). As with the present study, these prior investigations are observational in design and therefore cannot establish causality; the associations they report, like those presented here, should be interpreted as hypothesis-generating rather than confirmatory of a causal protective effect.

Despite this evidence, no published study has directly examined the association between the NNR score and T2DM risk, either globally or within the Saudi population specifically. Prior Saudi-based dietary research on T2DM has instead relied predominantly on pattern-adherence indices, most notably the Mediterranean Diet Score, or on food-group and single-nutrient analyses (13, 14); to date, no study has applied a nutrient-density-based composite index such as the NNR score, which captures micronutrient adequacy independent of adherence to any single culturally specific dietary pattern, to this population. Furthermore, the distinctive dietary environment of Saudi Arabia shaped by a cultural tradition that combines high consumption of refined carbohydrates, red meats, and sugar-sweetened beverages with traditional plant-based foods necessitates context-specific investigation that cannot be generalised from Western or East Asian cohorts (4, 5). Therefore, the present study aimed to investigate the association between the NNR score and the risk of T2DM among Saudi adults using a hospital-based case–control design, with the expectation that its findings will inform evidence-based dietary recommendations and public health strategies for T2DM prevention in this high-burden setting.

## Materials and methods

### Study population

This hospital-based case–control study was conducted at Prince Abdulaziz bin Musaed Hospital, Arar, Saudi Arabia, between November 2024 and August 2025. A total of 420 participants were enrolled, comprising 140 cases with a confirmed diagnosis of T2DM and 280 non-diabetic controls matched to cases in a 1:2 ratio by age (18–60 years) and sex. Case group inclusion criteria required a T2DM diagnosis confirmed within the 6 months preceding enrolment, based on standard glycaemic criteria consistent with American Diabetes Association (ADA) guidelines: a fasting plasma glucose (FPG) ≥ 126 mg/dL on two separate occasions, and/or a two-hour post-load plasma glucose (2 h-PG) ≥ 200 mg/dL during a 75 g oral glucose tolerance test (OGTT), and/or a glycated haemoglobin (HbA1c) ≥ 6.5% (15). Cases were required to be adults aged 18–60 years and willing to provide written informed consent. Control group inclusion criteria required documented normal glucose tolerance, defined as an FPG < 100 mg/dL and a 2 h-PG < 140 mg/dL on OGTT, with no prior diagnosis of any form of diabetes mellitus (15). Controls were recruited from the same healthcare facility to minimise selection bias. Exclusion criteria applied to both groups included: (1) a confirmed diagnosis of type 1 diabetes mellitus or gestational diabetes; (2) clinically significant chronic comorbidities likely to confound dietary assessment or metabolic outcomes, including end-stage renal disease, hepatic cirrhosis, malabsorptive gastrointestinal disorders, or active malignancy; (3) use of medications known to affect glucose metabolism or dietary intake, such as systemic corticosteroids, antipsychotics, or weight-loss pharmacotherapy; (4) adherence to a medically prescribed or restrictive dietary regimen at the time of enrolment; (5) current pregnancy or lactation; and (6) inability or unwillingness to complete the dietary assessment instrument. To reduce the influence of dietary misreporting, participants whose reported total daily energy intake fell outside a physiologically plausible range defined as <800 kcal/day or >4,200 kcal/day were excluded from the final analyses (16).

### Dietary assessment

Dietary intake was evaluated using a validated semi-quantitative food frequency questionnaire (FFQ) containing 152 food and beverage items (17). To reflect pre-diagnosis dietary habits while reducing recall bias, case participants were asked to report their habitual food consumption during the 12 months before their T2DM diagnosis, while control participants reported their dietary intake during the year preceding the interview date. Consumption frequency was recorded on daily, weekly, or monthly intervals and subsequently standardised to average daily intake, with monthly frequencies converted based on a 30.5-day reference month. Serving sizes were estimated using standardised household measurements, and the daily gram-equivalent of each food item was derived by multiplying the serving size by the corresponding daily frequency. Daily intake of energy, macronutrients, and micronutrients was calculated using Nutritionist IV dietary analysis software (18). To maintain data quality and completeness, all completed questionnaires were reviewed by trained interviewers immediately after administration. To minimise recall bias, all interviews were conducted by trained interviewers using standardised probing techniques and visual food-portion aids, and both groups were interviewed within a comparable timeframe relative to their respective reference period. Nonetheless, because dietary recall in cases was anchored to the period preceding a since-confirmed T2DM diagnosis, differential recall cannot be entirely excluded and is addressed further in the Limitations section.

### Anthropometric, dietary, and physical activity assessment

In addition to dietary intake, anthropometric measurements and physical activity levels were assessed for all participants. Sociodemographic information including age, sex, marital status, ethnicity, and smoking status was collected through structured face-to-face interviews. Height and weight were measured by trained personnel using a calibrated SECA stadiometer and weighing scale, with measurement precision of 0.5 cm and 100 g, respectively. Body mass index (BMI) was computed by dividing each participant’s weight in kilogrammes by the square of their height in metres (kg/m^2^). Physical activity level was assessed using the International Physical Activity Questionnaire (IPAQ) (19).

### Assessment of the naturally nutrient rich score

The NNR score is derived from a nutrient-to-calorie ratio framework that quantifies micronutrient content relative to established dietary guidelines (6). To calculate the NNR Score, the daily value (DV) for each nutrient was determined per 2,000 kcal of energy intake, based on the US Dietary Reference Intakes (DRIs). Specifically, the habitual dietary intake of 14 nutrients comprising protein, vitamins A, C, D, E, B1, B2, and B12, calcium, zinc, iron, folate, potassium, and unsaturated fatty acids was compared against the reference intake levels recommended for a 2,000-calorie diet by the Food and Nutrition Board. Once the DV for each nutrient was established, the NNR score was computed as the mean percentage DV across all 14 nutrients, as expressed in the following formula:

NNRscore=Σ(%DVper2,000kcal)/14.

### Statistical analysis

Differences in daily nutrient intake between case and control groups were examined using the independent samples t-test. To evaluate the association between diabetes and the NNR score, binomial logistic regression analysis was conducted across three sequential models: an unadjusted model (Model 1); a model controlling for age, sex, physical activity and smoking status (Model 2); and a fully adjusted model that additionally accounted for BMI and total caloric intake (Model 3). All statistical analyses were carried out using SPSS software version 23.0 (SPSS Inc., Chicago, IL, USA), with a two-sided *p*-value of less than 0.05 defined as the threshold for statistical significance.

## Results

Table 1 presents the general characteristics of the study participants. A total of 140 cases and 280 controls were included in this study. The two groups were comparable in terms of age, sex, employment status, smoking prevalence, educational level, and physical activity (all *p* > 0.05). However, cases had a significantly higher mean BMI compared to controls (28.32 ± 2.64 vs. 26.85 ± 2.15 kg/m^2^; *p* = 0.039), which was the only baseline characteristic that differed significantly between the two groups.

**Table 1:** General characteristics of study participants.

Variables	Cases (*N* = 140)	Controls (*N* = 280)	*p*-value
Age (year)	51.28 ± 11.06	49.5 ± 10.82	0.121
Body mass index (kg/m^2^)	28.32 ± 2.64	26.85 ± 2.15	0.039
Males n (%)	71 (50.71)	144 (51.42)	0.354
Employment n (%)	80 (57.14)	173 (61.79)	0.241
Smokers n (%)	8 (5.71)	15 (5.31)	0.134
Education (Illiterate & primary) n (%)	82 (58.57)	150 (53.57)	0.091
Low activity level n (%)	124 (88.57)	229(81.78)	0.064

Data are presented as mean ± standard deviation or Number (%). Independent sample t-test or Mann–Whitney u-test were used for comparison of quantitative variables. Chi-square test or Fisher’s exact test were used for comparison of qualitative variables.

Table 2 presents the comparison of daily nutrient intake between case and control groups. Cases had significantly higher caloric intake (2796.35 ± 179.14 vs. 2571.72 ± 407.21 kcal/day; *p* = 0.028), carbohydrate (371.24 ± 35.91 vs. 375.85 ± 53.67 g/day; *p* = 0.031), and cholesterol (276.03 ± 55.81 vs. 260.13 ± 63.29 g/day; *p* = 0.019). PUFA intake was significantly lower in cases compared to controls (18.37 ± 4.61 vs. 19.50 ± 4.37 g/day; *p* = 0.009), whereas oleic acid was significantly higher among cases (11.17 ± 5.63 vs. 8.55 ± 3.29 g/day; *p* = 0.001). Cases also consumed significantly less fibre (26.73 ± 4.99 vs. 29.00 ± 6.29 g/day; *p* = 0.001), crude fibre (*p* = 0.012), and sugar (119.02 ± 20.81 vs. 129.75 ± 32.74 g/day; *p* = 0.001). Among micronutrients, cases had significantly lower intakes of magnesium (*p* = 0.012), manganese (*p* = 0.004), selenium (*p* = 0.001), fluoride (*p* = 0.001), vitamin A (*p* = 0.001), beta-carotene (*p* = 0.001), vitamin E (*p* = 0.001), vitamin B₁ (*p* = 0.033), folate (*p* = 0.001), and biotin (*p* = 0.003), while vitamin K intake was significantly higher in cases than controls (164.69 ± 51.23 vs. 146.97 ± 27.38 mg/day; *p* = 0.001). No significant differences were observed in protein, fat, SFA, MUFA, EPA, DHA, soluble and insoluble fibre, iron, calcium, phosphorus, zinc, copper, sodium, potassium, or most B vitamins between the two groups.

**Table 2:** Comparison of dietary intake of nutrients among the case and control groups.

Variable	Cases (*n* = 140) Mean ± SD	Controls (*n* = 280) Mean ± SD	*p* value
Calorie (kcal/day)	2796.35 ± 179.14	2571.72 ± 157.21	0.028
Protein (g/day)	88.73 ± 9.43	88.36 ± 20.12	0.841
Carbohydrate (g/day)	371.24 ± 35.91	357.85 ± 53.67	0.031
Fat (g/day)	91.86 ± 10.84	93.11 ± 20.38	0.318
Cholesterol (g/day)	276.03 ± 55.81	260.13 ± 63.29	0.019
SFA (g/day)	34.64 ± 6.22	34.15 ± 10.21	0.601
MUFA (g/day)	31.13 ± 4.96	31.75 ± 6.51	0.317
PUFA (g/day)	18.37 ± 4.61	19.50 ± 4.37	0.009
Oleic acid (g/day)	11.17 ± 5.63	8.55 ± 3.29	0.001
Linolenic acid (g/day)	3.79 ± 0.83	3.95 ± 0.76	0.057
Linoleic acid (g/day)	9.98 ± 4.21	9.02 ± 3.57	0.084
EPA (g/day)	3.22 ± 0.27	3.24 ± 0.28	0.312
DHA (g/day)	3.64 ± 0.58	3.59 ± 0.61	0.427
Fibre (g/day)	26.73 ± 4.99	29.00 ± 6.29	0.001
Soluble fibre (g/day)	3.95 ± 0.67	4.04 ± 0.81	0.238
Insoluble fibre (g/day)	8.04 ± 1.93	8.24 ± 2.44	0.389
Crude fibre (g/day)	12.04 ± 2.55	12.81 ± 3.57	0.012
Sugar (g/day)	119.02 ± 20.81	129.75 ± 32.74	0.001
Iron (mg/day)	16.67 ± 3.32	16.58 ± 2.94	0.779
Calcium (mg/day)	1209.92 ± 121.04	1233.03 ± 501.17	0.597
Magnesium (mg/day)	334.99 ± 44.07	351.84 ± 74.83	0.012
Phosphors (mg/day)	1399.32 ± 175.03	1415.61 ± 463.20	0.687
Zinc (mg/day)	13.71 ± 2.57	13.55 ± 3.56	0.648
Copper (mg/day)	2.52 ± 0.68	2.64 ± 0.72	0.117
Manganese (mg/day)	7.79 ± 1.87	8.20 ± 1.12	0.004
Selenium (mg/day)	54.50 ± 26.13	71.66 ± 20.26	0.001
Fluoride (mg/day)	1586.28 ± 9790.41	10823.77 ± 3434.34	0.001
Chromium (μg/day)	3.08 ± 0.19	3.95 ± 0.17	0.157
Molybdenum (μg/day)	53.85 ± 9.52	53.71 ± 4.83	0.878
Sodium (mg/day)	6357.99 ± 1526.86	6087.99 ± 1062.45	0.031
Potassium (mg/day)	3924.20 ± 391.39	3993.72 ± 888.00	0.287
Vitamin A (μg/day)	694.04 ± 161.00	845.43 ± 291.04	0.001
Beta-carotene (μg/day)	2079.26 ± 594.72	2471.03 ± 939.43	0.001
Vitamin E (mg/day)	13.11 ± 4.29	16.06 ± 5.46	0.001
Alpha-tocopherol (mg/day)	12.10 ± 5.15	12.02 ± 3.87	0.208
Vitamin B₁ (mg/day)	4.96 ± 0.89	5.15 ± 0.87	0.033
Vitamin B₂ (mg/day)	4.986 ± 1.02	5.33 ± 1.21	0.338
Vitamin B₃ (mg/day)	24.40 ± 2.66	24.71 ± 3.27	0.327
Vitamin B₆ (mg/day)	4.89 ± 0.83	4.88 ± 0.87	0.888
Folate (μg/day)	519.41 ± 98.75	574.01 ± 82.47	0.001
Vitamin B₁₂ (mg/day)	7.43 ± 1.93	7.23 ± 2.59	0.408
Pentatonic acid (mg/day)	8.47 ± 1.80	8.32 ± 1.96	0.438
Biotin (μg/day)	29.86 ± 4.71	31.72 ± 8.17	0.003
Vitamin C (mg/day)	148.12 ± 22.88	153.93 ± 57.88	0.248
Vitamin D (mg/day)	1.12 ± 0.85	1.25 ± 1.91	0.178
Vitamin K (mg/day)	164.69 ± 51.23	146.97 ± 27.38	0.001

DHA, docosahexaenoic acid; EPA, eicosapentaenoic acid; MUFA, monounsaturated fatty acid; PUFA, polyunsaturated fatty acid; SFA, saturated fatty acid.

Table 3 presents the comparison of daily nutrient intake between participants with higher and lower NNR scores (cut-off: 99.53). Participants with higher NNR scores had significantly greater intakes of fibre (30.21 ± 5.41 vs. 28.34 ± 6.54 g/day; *p* = 0.003), crude fibre (*p* = 0.033), magnesium (*p* = 0.042), zinc (15.42 ± 2.78 vs. 13.76 ± 3.78 mg/day; *p* = 0.001), and potassium (*p* = 0.042) compared to those with lower NNR scores. Furthermore, intakes of beta-carotene (*p* = 0.006), vitamin E (*p* = 0.001), vitamins B₁, B₂, and B₁₂ (all *p* < 0.05), biotin (*p* = 0.008), vitamin C (*p* = 0.001), and vitamin D (*p* = 0.006) were all significantly higher in the higher NNR group. In contrast, no statistically significant differences were observed between the two groups in caloric intake, macronutrients including protein, carbohydrate, fat, and cholesterol, most fatty acids, or the remaining micronutrients such as iron, calcium, selenium, sodium, and folate (all *p* > 0.05).

**Table 3:** Comparison of daily nutrient intake by NNR score level (Low NNR: Score ≤99.53; High NNR: Score >99.53, based on the sample median).

Variable	Higher than 99.53 Mean ± SD	Lower than 99.53 Mean ± SD	*p* value
Calorie (kcal/day)	2556.00 ± 226.84	2539.94 ± 488.56	0.681
Protein (g/day)	90.78 ± 10.87	88.38 ± 24.63	0.213
Carbohydrate (g/day)	369.45 ± 37.42	366.54 ± 61.78	0.572
Fat (g/day)	93.91 ± 11.73	94.18 ± 25.07	0.903
Cholesterol (g/day)	268.41 ± 42.65	260.70 ± 81.34	0.234
Saturated fat (g/day)	35.23 ± 6.04	35.45 ± 12.52	0.834
MUFA (g/day)	32.69 ± 4.52	32.38 ± 7.91	0.637
PUFA (g/day)	20.20 ± 3.74	20.21 ± 5.39	0.983
Oleic (g/day)	10.18 ± 3.19	10.41 ± 5.19	0.614
Linoleic (g/day)	10.17 ± 3.21	10.37 ± 4.60	0.624
Linolenic (g/day)	4.94 ± 0.82	4.93 ± 0.77	0.172
EPA (g/day)	4.23 ± 0.27	4.20 ± 0.26	0.374
DHA (g/day)	4.63 ± 0.61	4.53 ± 0.59	0.097
Fibre (g/day)	30.21 ± 5.41	28.34 ± 6.54	0.003
Soluble fibre (g/day)	5.00 ± 0.75	5.95 ± 0.82	0.362
Insoluble fibre (g/day)	9.19 ± 1.92	9.18 ± 2.73	0.978
Crude fibre (g/day)	14.07 ± 2.88	13.19 ± 3.86	0.033
Sugar (g/day)	129.27 ± 20.53	125.30 ± 39.14	0.204
Iron (mg/day)	22.65 ± 2.61	22.50 ± 3.52	0.624
Calcium (mg/day)	1240.20 ± 148.97	1216.81 ± 641.10	0.624
Magnesium (mg/day)	353.14 ± 45.12	339.46 ± 84.23	0.042
Phosphorus (mg/day)	1426.46 ± 175.82	1394.53 ± 591.80	0.463
Zinc (mg/day)	15.42 ± 2.78	13.76 ± 3.78	0.001
Copper (mg/day)	1.94 ± 0.748	1.83 ± 0.641	0.087
Manganese (mg/day)	9.00 ± 1.57	9.04 ± 1.83	0.803
Selenium (mg/day)	68.04 ± 13.69	65.71 ± 29.21	0.314
Fluoride (mg/day)	12090.22 ± 5034.87	12604.93 ± 8867.92	0.473
Chromium (μg/day)	4.05 ± 0.16	4.87 ± 0.15	0.187
Molybdenum (μg/day)	53.94 ± 7.43	54.74 ± 7.83	0.913
Sodium (mg/day)	6187.52 ± 896.14	6156.11 ± 1694.06	0.824
Potassium (mg/day)	4056.45 ± 583.16	3886.23 ± 1047.23	0.042
Vitamin A (μg/day)	753.40 ± 177.43	730.63 ± 290.21	0.334
Beta-carotene (μg/day)	2484.57 ± 831.47	2221.99 ± 1065.02	0.006
Vitamin E (mg/day)	17.31 ± 4.06	15.17 ± 6.62	0.001
Alpha-tocopherol (mg/day)	13.16 ± 3.33	13.71 ± 5.34	0.394
Vitamin B₁ (mg/day)	6.24 ± 0.82	5.91 ± 0.87	0.002
Vitamin B₂ (mg/day)	7.62 ± 1.03	5.92 ± 1.22	0.002
Vitamin B₃ (mg/day)	25.57 ± 2.68	25.60 ± 3.43	0.934
Vitamin B₆ (mg/day)	4.94 ± 0.84	5.85 ± 0.88	0.492
Folate (mg/day)	564.74 ± 73.74	550.94 ± 117.07	0.163
Vitamin B₁₂ (mg/day)	8.86 ± 1.79	7.70 ± 2.92	0.001
Pantothenic acid (mg/day)	9.49 ± 1.75	9.19 ± 2.18	0.118
Biotin (μg/day)	33.05 ± 0.71	31.04 ± 8.82	0.008
Vitamin C (mg/day)	163.11 ± 66.49	143.16 ± 35.97	0.001
Vitamin D (mg/day)	1.85 ± 0.76	1.02 ± 0.73	0.006
Vitamin K (mg/day)	156.64 ± 860.94	150.97 ± 46.12	0.224

DHA, docosahexaenoic acid; EPA, eicosapentaenoic acid; MUFA, monounsaturated fatty acid; NNR, naturally nutrient rich; PUFA, polyunsaturated fatty acid.

Table 4 presents the association between NNR score and the risk of T2DM across three logistic regression models. In the crude model (Model 1), a higher NNR score was significantly associated with reduced odds of T2DM (OR = 0.93; 95% CI: 0.87–0.96; *p* < 0.001). This inverse association remained statistically significant after adjustment for age, sex, smoking, and physical activity in Model 2 (OR = 0.82; 95% CI: 0.71–0.88; *p* < 0.001). Furthermore, following additional adjustment for BMI and caloric intake in the fully adjusted model (Model 3), the protective association persisted (OR = 0.90; 95% CI: 0.82–0.93; *p* < 0.001). Across all three models, higher NNR scores were consistently and significantly associated with lower odds of T2DM, suggesting that a more nutrient-rich diet may play a protective role against the development of type 2 diabetes.

**Table 4:** Odds ratios (95% CI) for T2DM associated with a one-unit increase in NNR score, across adjusted logistic regression models.

Variable	OR	95% CI	*p* value
Model 1	0.93	0.87 to 0.96	*p* < 0.001
Model 2	0.82	0.71 to 0.88	*p* < 0.001
Model 3	0.90	0.82 to 0.93	*p* < 0.001

Model 1: crude. Model 2: adjusted for age and sex, smoking and physical activity. Model 3: additional adjustments for BMI and calorie intake.

## Discussion

To our knowledge, this is the first case–control study to directly examine the association between the Naturally Nutrient Rich (NNR) score and the risk of type 2 diabetes mellitus (T2DM) in a Saudi adult population. The principal finding of the present study was that a higher NNR score reflecting greater dietary nutrient density relative to energy intake was independently and inversely associated with T2DM risk across all three logistic regression models, including after full adjustment for age, sex, smoking, physical activity, BMI, and total caloric intake (OR = 0.90; 95% CI: 0.82–0.93; *p* < 0.001). These findings provide novel epidemiological evidence that a nutrient-dense dietary pattern, as quantified by the NNR score, was associated with lower odds of T2DM (6, 7).

The inverse association between the NNR score and T2DM risk observed in the present study is consistent with the broader evidence base linking overall dietary quality to reduced metabolic disease risk. Rivas-Garcia et al. (11), in a prospective analysis of the CORDIOPREV cohort, demonstrated that greater adherence to nutrient-rich food patterns was associated with a more than 50% reduction in incident T2DM risk among patients with coronary heart disease (11). Similarly, El-Sehrawy et al. reported, in a case–control design analogous to the present study, that higher scores on the Diet Quality Index–International and its revised counterpart were significantly associated with lower odds of T2DM (12). Aligned with these observations, a large prospective cohort study by Fung et al. demonstrated that dietary patterns characterised by high intakes of whole grains, vegetables, fruits, legumes, and fish all foods contributing favourably to the NNR score were associated with a significantly reduced incidence of T2DM over a follow-up period of 14 years (20). Furthermore, Schulze et al. reported in the EPIC-Potsdam cohort that dietary quality indices emphasising micronutrient adequacy were inversely associated with T2DM incidence, independent of BMI and physical activity (21). The consistency of our findings with these independent investigations, despite differences in study population, dietary assessment instrument, and the specific index employed, strengthens confidence that high dietary nutrient density is consistently associated with lower T2DM risk across diverse populations and dietary assessment methods, although the observational nature of all cited studies precludes causal inference.

The micronutrient-level findings provide biologically plausible, though indirect, support for this association; as no biomarkers of nutrient status were measured in this study, the mechanisms discussed below are proposed on the basis of prior literature rather than demonstrated directly by our data. Cases had significantly lower intakes of magnesium, selenium, folate, vitamins A, E, and B₁, beta-carotene, and biotin relative to controls (all *p* < 0.05). Several of these micronutrients have established roles in glucose–insulin homeostasis: magnesium and zinc are required cofactors for insulin signalling and beta-cell insulin storage (22–24); selenium and vitamin E function as antioxidants that limit oxidative damage to pancreatic beta cells (25, 26); folate contributes to one-carbon metabolism implicated in epigenetic regulation of glucose metabolism (22); and vitamin A/beta-carotene modulate inflammatory pathways relevant to insulin action (27). Zinc is an essential structural component of insulin hexamers within pancreatic beta-cell secretory granules, and adequate zinc status is required for normal insulin synthesis, storage, and secretion (24). Collectively, these observations suggest that the inverse association between a high NNR score and T2DM risk may be attributable, at least in part, to the combined adequacy of multiple micronutrients.

The macronutrient profile of cases further supports the plausibility of this association: cases reported higher caloric and cholesterol intake alongside lower fibre and PUFA intake than controls. Higher dietary fibre intake has been associated with reduced T2DM incidence in a large meta-analysis, through mechanisms including attenuated postprandial glucose absorption and favourable modulation of gut microbiota (28), while higher PUFA intake, including via substitution for saturated fat, has been shown in randomised feeding trials to improve insulin sensitivity (29, 30). These macronutrient differences are broadly concordant with dietary patterns previously identified as risk-modifying in Saudi and regional populations (4, 13), supporting the validity of the dietary assessment used here.

The present study is also the first to characterise the nutrient intake profile stratified by NNR score level in this population. Participants in the higher NNR stratum consumed significantly more dietary fibre, zinc, potassium, magnesium, and multiple vitamins relative to those in the lower NNR stratum, while macronutrient intakes including total energy, protein, fat, and carbohydrate did not differ significantly between the two groups. This pattern validates the theoretical premise of the NNR score: that it captures variance in micronutrient density independent of total energy intake, thereby identifying qualitative differences in diet composition that would be obscured by analyses restricted to macronutrient or total energy data alone (6, 7). Drewnowski and Fulgoni previously demonstrated the capacity of nutrient density metrics, including the NNR score, to discriminate between food groups on the basis of micronutrient adequacy rather than caloric content, and proposed their application as standardised tools for dietary guidance and food labelling (7). The ability of the NNR score to discriminate between nutrient-adequate and nutrient-poor dietary patterns within a population of comparable caloric intake has important implications for dietary assessment in clinical and public health contexts, particularly in populations such as Saudi Arabia where diets may be simultaneously energy-dense and micronutrient-insufficient (13, 14).

The elevated BMI observed among cases relative to controls in the present study (28.32 ± 2.64 vs. 26.85 ± 2.15 kg/m^2^; *p* = 0.039) is consistent with the well-established role of adiposity as a central risk factor for T2DM, and has been extensively documented in Saudi and regional epidemiological literature (5). Adipose tissue-derived pro-inflammatory adipokines, including tumour necrosis factor-alpha and interleukin-6, are known to impair insulin receptor substrate phosphorylation and downstream signalling cascades, thereby promoting peripheral insulin resistance (31). Notably, the association between the NNR score and T2DM risk in the present study persisted after adjustment for BMI (Model 3), indicating that the observed association between dietary nutrient density and T2DM risk operates through mechanisms that appear at least partially independent of adiposity-mediated pathways. This finding is of particular clinical relevance, as it suggests that dietary quality interventions may reduce T2DM risk even in the absence of weight loss a consideration with important implications for public health messaging in high-obesity settings.

From a theoretical standpoint, the present findings align with the nutrient density hypothesis of chronic disease prevention, which posits that diets supplying adequate micronutrients relative to energy intake promote metabolic resilience by sustaining antioxidant defences, reducing low-grade systemic inflammation, and preserving mitochondrial function all of which are implicated in the pathogenesis of insulin resistance (10). From a public health perspective, the NNR score offers practical advantages over more complex dietary indices for use in population screening and dietary counselling in Saudi Arabia, where validated, culturally adapted dietary quality tools remain scarce (13, 14). The computation of the NNR score from standard dietary assessment data without requiring extensive dietitian interpretation suggests potential future suitability for integration into clinical practice and large-scale nutritional surveillance systems, pending the external validation discussed below.

Several limitations of the present study warrant consideration when interpreting its findings. First, the case–control design precludes the establishment of temporal sequence between dietary exposure and disease onset, and therefore causal inference cannot be drawn. Second, dietary assessment relied on a self-reported FFQ, which is subject to measurement error, social desirability bias, and limitations in the precision of portion size estimation. These limitations are particularly relevant to micronutrient estimates, which typically show weaker validity and reproducibility in FFQ-based assessments than macronutrient or total energy estimates, owing to greater variability in food-composition database values and less consistent recall of foods contributing small absolute quantities of specific micronutrients. Third, recall bias is an inherent limitation of any retrospective dietary exposure assessment; although case participants were instructed to recall their habitual diet prior to diagnosis, the influence of post-diagnosis dietary modification on reported intake cannot be fully excluded. This raises the possibility of reverse causation, whereby the disease process or its diagnosis influenced reported nutrient intake. Fourth, while the study adjusted for major potential confounders including BMI, smoking, physical activity, and total caloric intake, residual confounding from unmeasured variables such as socioeconomic status, family history of diabetes, sleep quality, psychological stress, gut microbiome composition, and use of dietary supplements remains possible (32). Fifth, both cases and controls were recruited from a single hospital. This design, while minimising some sources of heterogeneity, introduces potential selection bias: individuals attending this particular facility may not be representative of the broader Saudi population in terms of socioeconomic status, health-seeking behaviour, or regional dietary patterns. This limits the generalisability of our findings beyond this specific hospital catchment population and region, and multi-centre studies are needed to confirm external validity.

Future research should seek to address these limitations through prospective cohort designs with repeated dietary assessments that can establish temporal directionality and support causal inference. Randomised controlled trials examining the effect of dietary interventions designed to increase NNR scores on glycaemic markers and incident T2DM would provide the highest level of evidence. Studies incorporating objective biomarkers of nutrient status such as serum micronutrient concentrations, inflammatory markers, and measures of insulin resistance alongside dietary data would strengthen the mechanistic evidence base (33). Additionally, multi-centre studies recruiting participants from diverse regions and socioeconomic strata within Saudi Arabia and the broader MENA region are needed to establish the external validity of the present findings. Finally, investigation of potential effect modification by genetic polymorphisms in nutrient metabolism pathways, such as variants in the SLC30A8 gene encoding a zinc transporter expressed in pancreatic beta cells, may identify subpopulations at differential risk who could benefit most from targeted dietary quality interventions (24). The NNR score is a simple and practical tool for dietary assessment, its routine clinical use in Saudi Arabia would require further validation in larger and more diverse populations across different regions of the country, as well as standardisation of dietary assessment methods. In our study, the NNR score was analysed primarily as a dichotomous variable based on the median. While this approach provides a clear and clinically interpretable comparison between high and low micronutrient density groups, we acknowledge that it may not fully capture the dose–response relationship between the NNR score and T2DM risk. Future studies with larger sample sizes are warranted to explore this association using quantile-based categorisation or restricted cubic spline analyses to better characterise the shape of the relationship and identify potential threshold effects.

## Conclusion

This hospital-based case–control study found that a higher NNR score, reflecting greater dietary nutrient density, was associated with reduced odds of T2DM among Saudi adults, independent of age, sex, smoking, physical activity, BMI, and total caloric intake. As an observational study, these findings establish an association rather than a causal relationship, and should be interpreted as hypothesis-generating. These results support the potential utility of the NNR score as a nutrient-density-based dietary quality metric in the Saudi context. Before wider adoption, however, external validation of the NNR score in larger and more diverse Saudi populations is warranted, alongside prospective cohort and interventional studies to confirm these findings, establish temporal and causal relationships, and evaluate the efficacy of NNR-targeted dietary interventions in reducing T2DM incidence.

## Data Availability

The raw data supporting the conclusions of this article will be made available by the authors, without undue reservation.
